# Assessment of the Possibility of Using Fly Ash from Biomass Combustion for Concrete

**DOI:** 10.3390/ma14216708

**Published:** 2021-11-07

**Authors:** Jakub Jura, Malgorzata Ulewicz

**Affiliations:** Faculty of Civil Engineering, Czestochowa University of Technology, Dabrowskiego 69 Street, PL 42-201 Czestochowa, Poland; jakub.jura@pcz.pl

**Keywords:** concrete, mechanical properties, low-temperature resistance, fly ash, biomass

## Abstract

This article analyses the possibility of using fly ash from the combustion of wood–sunflower biomass in a fluidized bed boiler as an additive to concrete. The research shows that fly ash applied in an amount of 10–30% can be added as a sand substitute for the production of concrete, without reducing quality (compression strength and low-temperature resistance) compared to control concrete. The 28-day compressive strength of concrete with fly ash increases with the amount of ash added (up to 30%), giving a strength 28% higher than the control concrete sample. The addition of fly ash reduces the extent to which the compression strength of concrete is lowered after low-temperature resistance tests by 22–82%. The addition of fly ash in the range of 10–30% causes a slight increase in the water absorption of concrete. Concretes containing the addition of fly ash from biomass combustion do not have a negative environmental impact with respect to the leaching of heavy metal ions into the environment.

## 1. Introduction

The development of industry causes a systematic increase in the amount of generated waste. Some of the wastes produced, such as fly ashes and slags from the combustion of conventional fuels (hard and brown coal), are used to produce composite materials with a cement matrix, such as cement mortars or concretes [1,2,3,4,5,6,7,8,9,10]. In recent years, there have also been a number of reports in the literature on the possibility of using waste from construction ceramics [11,12,13], sanitary and household ceramics [14,15,16,17,18], glass cullet [19,20,21,22] and polymer materials [23,24,25] to produce cement mortars and concretes.

There have also been reports of the possibility of using fly ashes from the co-combustion of hard coal and biomass in conventional or fluidized bed boilers for this purpose. Mortars and concretes with the addition of such ash usually achieve similar or lower strength values after 28 days of maturation (75% of the control samples [26], 98–84% [27], 72–93% [28], 98–46% [29]), and after a longer period (90–180 days) they increase their compressive strength, ultimately achieving a strength similar to [26,29,30] or higher than the control samples (2–20% higher than control samples [27], 5–12% [29]. The results obtained by the authors of these studies confirm that the ashes produced in co-combustion processes have a higher reactivity and can be a useful raw material in the production of cement matrix materials [29]. Currently, the physical and chemical properties of the ashes generated during combustion process are being tested, e.g., forest residues, the pulp and paper industry, sugar cane or corn cobs, and attempts are being made to develop methods for their management in various sectors of the economy [31,32,33,34,35,36].

There are few reports in the literature on the laboratory use of ashes from biomass combustion, including the production of composite materials with a cement matrix [37]. Most of the studies available in the literature concern the properties of ash and the possible use of fly ash from the combustion of sugar cane bagasse, most often used in the amount of 5–30% of the cement mass [38,39,40,41]. Reports show that the addition of such ash may both positively and negatively affect the mechanical and physical properties of materials with a cement matrix. The compressive strength of materials with such additives decreased, depending on the type of biomass used and the amount of fly ash added. Compressive strength was lower than the control samples (5–25% [42], 18% [43], 55% [44], 25% [45]) or higher than the control samples (3–14% [42], 30% [43], 5% [44], 1–17% [45], 17% [46], 13% [47]). The best results in terms of compressive strength were achieved by samples containing ashes from wood in the amount of 5% [42], 10% [44] and 20% [45], and in the case of sugar cane bagasse at 5–10% [38,39,40,41,43,47,48]), while the worst results were for samples containing ashes from wood in greater proportions (15% [42], 20% [43], 25% [45]) and for ash from the combustion of sugar cane bagasseused in proportions of 20–25% [38,39,40,41,43,47,48].

Mortars containing up to 30% ash usually showed higher resistance to freezing and thawing than the control samples (reduction of the drop in compressive strength up to 95% [46], down to 50% [47]). Currently, fluidized ashes generated during biomass combustion in fluidized bed boilers (classified as waste with the code 10 01 82), due to the different physicochemical properties determined by the diversity of incinerated biomass, are not usable and are therefore deposited in landfills, which places a burden on the natural environment. For these reasons, it is advisable to develop methods of managing these types of ashes. Therefore, this study attempts to evaluate the possibility of using waste fly ash from the combustion of biomass itself in a fluidized bed boiler with a circulating bed for the production of composite building materials with a cement matrix. Such a method of using this waste would significantly reduce the usage of natural resources and limit the negative environmental impact of waste ash.

## 2. Materials and Methods

### 2.1. Materials

Fly ash from the combustion process in a boiler with a circulating fluidized bed of biomass consisting of 80% waste wood and 20% sunflower was used for the research. Fly ash came from a power plant (GDF SUEZ, Połaniec, Poland) in the Świętokrzyskie Voivodeship and was tested in accordance with PN EN 450 1:2012. Ash roasting losses amounted to 2.9%. According to the PN-EN 451-2:2017-06 standard, the fineness of the samples as a residue on the 0.045 mm sieve is 7.9%, and the fly ash density is 2.35 g/cm^3^. The elemental composition of the fly ash made with the use of the XRF X-ray spectrometer (Thermo Fisher Scientific, Waltham, MA, USA) is presented in Table 1.

**Figure 1:**
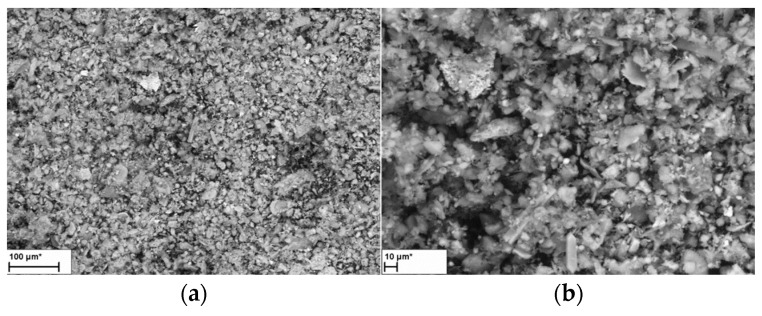
Fly ash microstructure in magnification: (**a**) 400×; (**b**) 1000×.

Fly ash consists mainly of silicon oxide (50.2%), aluminium oxide (12%), calcium oxide (11.82%) and almost 8% potassium oxide. The remaining compounds constitute less than 4%, which traces include Zn, Ba, CR, Sr, Zr, Cu, Rb, Ni and Pb, among other materials. The microstructure of the fly ash used for the tests (determined with an LEO Electron Microscopy Ltd. apparatus, Cambridge, UK) is presented in Figure 1, and reveals that the fly ash grains have an inhomogeneous structure with sharp edges, characteristic of crushed stone aggregate.

**Figure 2:**
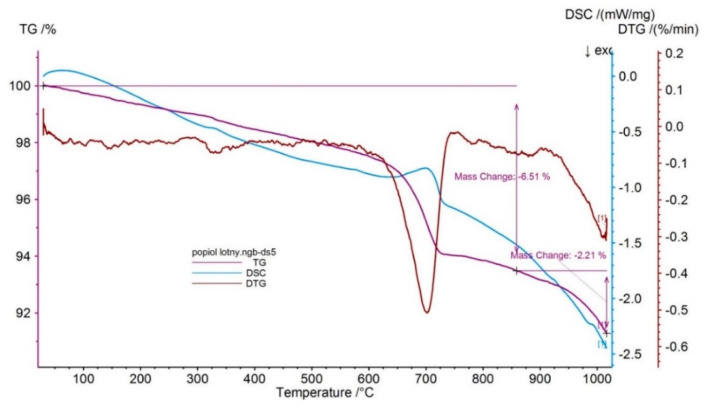
TGA-DTA thermogram for fly ash.

The TGA-DTA thermal analysis was also conducted for the fly ash used in the research (Figure 2).

The test was carried out in a Jupiter STA 449 F5 (Netzsch, Selb, Germany) in the range from 30 to 1000 °C with a temperature increase rate of 10 °C/min in air with a gas flow rate of 100 cm^3^/min. Up to a temperature of approximately 100 °C, the fly ash sample lost approximately 0.3% weight, which is related to the evaporation of water. At a temperature of about 350 °C, a peak was noticeable, which may have resulted from the transformation of Fe compounds. The peak was seen at a temperature of about 580–600 °C and corresponded to the conversion of α-quartz to β-quartz and the reaction between unreacted particles and activator trapped in the pores. Decomposition of aluminum hydroxide (Al(OH)_3_) also took place in the same temperature range. At temperatures of 700 °C, endothermic or exothermic reactions occurred, which corresponded to the decomposition of CaCO_3_, Ti(OH)_4_ and Mg(OH)_2_. The exothermic effect at about 900 °C may be related to the crystallization of amorphous ash phases. Observed changes were similar to those presented in [49] for a fly ash-based geopolymer. At a temperature of up to 900 °C, the loss of the sample mass (up to 6.51%) was related to the stripping of volatile parts. At temperatures above 900 °C, coal residues were burnt, and the fly ash sample lost more than 1% of its weight. The total weight loss was 8.7% in this temperature range. The DTG analysis also showed that in the case of the tested ash, the mass loss during heating was steady, and the greatest amplitude deviating from the entire line of the graph was for the temperature range of 650–800 °C. DSC Differential Scanning Calorimetry also enabled us to check the course of the formation of thermal effects in the sample. In the case of fly ash, the amount of heat obtained from the sample decreased almost steadily.

Two types of cements by CEMEX with high early strength were used for the tests: CEM I 42.5 R Portland cement and CEM II/A-V 42.5 R Portland ash cement.

Two types of coarse aggregate, i.e., gravel and basalt, as well as one type of fine aggregate, which was arenaceous quartz, were used for concrete tests. The composition of the control concrete with a gravel–sand (B1-0) and sand–basalt (B2-0) mixture of aggregates and CEM I cement, as well as that of a gravel–sand mixture of aggregates and CEM II per 1 m^3^ of concrete is presented in Table 2.

Table 3 presents the compositions of the designed concrete mixes containing fly ash. Fly ash was added to concrete in the amounts of 10%, 20%, 30% and 40% of the cement mass (used as a replacement for part of the sand calculated volumetrically), and the series of concretes were marked as B1-1, B1-2, B1-3 and B1-4, respectively, for the sand–gravel aggregates. In the case of sand–basalt aggregates, fly ash was added in the amounts of 10%, 20% and 30% of the cement mass, using ash as a substitute for part of the sand, and the series of concretes were marked as B2-1, B2-2 and B2-3. On the basis of the designed B1-0 control concrete, the members of the series were also made using CEM II/A-V 42.5 R ash cement, fly ash being used as a substitute for a part of the sand in the amounts of 10%, 20% and 30%, and were marked as B3-1, B3-2, B3-3, respectively.

### 2.2. Methods

Samples for the concrete mix tests were taken in accordance with the PN EN 12350 1:2011 standard. The air content in the concrete mixture was tested using the manometer method in accordance with the PN-EN 12350-7 standard.

Compressive strength tests of concretes were conducted (ToniTechnik 2030, Berlin, Germany) on 12 cubic samples with a side of 150 mm in accordance with PN EN 12390-3. A constant load speed of 1.0 MPa/s was chosen. The load was applied to the specimen and continuously increased until the highest load was obtained. After the test, the testing machine, based on the entered dimensions of the sample, gave the compressive strength in MPa.

Concrete water absorption was tested in accordance with PN B 06250:1988. The tests were performed on six cubic samples (each series) with sides of 150 mm. The samples were disassembled after 24 h and were kept in water for the next 7 days, and then the samples were kept in the air for 21 days. The tested concrete samples were placed in a vessel on a grate at a distance of 15 mm from the bottom of the vessel. After obtaining a constant mass, the samples were placed in a laboratory dryer and dried at 105 °C to a constant mass, i.e., until the next weighings showed differences below 0.2% of the mass of the samples. The water absorption by weight is defined as the ratio of the mass of water penetrating into the saturated material to its dry mass and is given as a percentage.

The low-temperature resistance of concrete was tested on the basis of the PN-B-06250:1988 standard using a Toropol K-010 chamber (Toropol, Warsaw, Poland). 12 samples with a side of 100 mm were taken from each series of concretes. Samples saturated with water were weighed and then subjected to frost resistance testing. Six samples were left in water at 18 ± 2 °C throughout the test, and the remaining six were wiped of water and weighed with an accuracy of 0.2%. These concrete samples were frozen in air at (−18 ± 2 °C) for 4 h and then thawed in water (at +18 ± 2 °C), also for 4 h. After 150 cycles of freezing and thawing the samples were reweighed and subjected to a compressive strength test.

Penetration of concrete with water under pressure was tested on the basis of the PN EN 12390-8 standard with the RatioTec WU60M apparatus (RatioTec, Essen, Germany), and three cubic samples with sides of 150 mm for each series were used for the tests. A water pressure of 500 kPa was applied to the samples for 72 h. After this time, the samples were taken out of the device and split in half. After the fracture surface had dried to a visible range of water, the maximum depth of water penetration was measured with an accuracy of 1 mm.

As part of the tests, a leaching analysis was also conducted based on the PN-EN-12457-2:2006 standard. For the test, samples containing 95% of grains smaller than 4 mm were prepared. For this purpose, the concrete samples were crushed in a jaw crusher and passed through a sieve with a mesh size of 4 mm. From the sample prepared in this way, an analytical sample (S) weighing 0.090 ± 0.005 kg was taken and placed in a vessel (bottle), and then distilled water (L) with electrical conductivity of 0.45 mS/m in the amount of 0.9 dm3 was added to it. According to the standard, the ratio of the washing liquid (L) to the solid phase (S) should be 10 dm^3^ to 1 kg. The closed vessel was placed in a mixing device (roller table) for 24 ± 0.5 h. The vessel was then allowed to sit for 15 ± 5 min to allow the suspension to settle. The effluent was filtered through a membrane filter with a pore diameter of 0.045 µm. The concentrations of selected metal ions were determined by induction plasma atomic emission spectrophotometry (with an ICP-AES spectrometer) and the effluent pH was determined. Two samples were tested from the selected series.

## 3. Results

### 3.1. Properties of Concrete Mixes

During the preparation of concrete mixes, samples were taken to test the consistency as well as the air content in the mixes. The fall of the cone for the B1-0 control concrete mix was 145 mm, which qualifies it as belonging to the S3 consistency class. The air content in this concrete mix was 3.4%. B1 concrete mixes containing fly ash used as part of the sand were denser than the B1-0 mix. The air content in the concrete mix decreased with the increase of the amount of added ash and amounted to 2.7% for the B1 mix containing 30% ash (Table 4). Mixes with the sand–basalt aggregate behaved similarly to the B1 mixes, too. The addition of fly ash to concrete mixes based on CEM II cement (B3 series) also caused the condensation of the concrete mix and a reduction of the air content.

### 3.2. The Impact of Ash Addition on the Compressive Strength of Concrete

**Figure 3:**
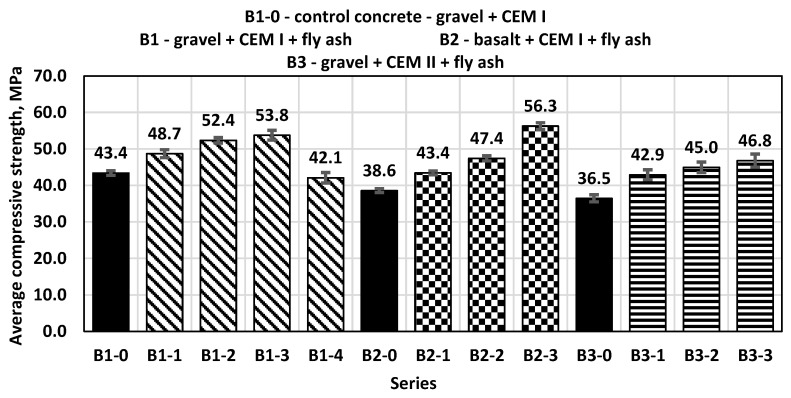
Average compressive strength after 7 days.

The concrete samples shaped into cubes with side dimensions of 150 mm were tested for compressive strength after 7, 28 and 56 days. After 7 days, the control concrete (B1-0) was marked by a compressive strength of 43.4 MPa. B1-0 concrete was the control concrete for the B1 series samples. All the concretes in the series with the addition of fly ash from biomass combustion in a fluidized bed boiler in the amounts of 10%, 20% and 30% of the weight of cement used instead of sand showed higher compressive strength after 7 days compared to the corresponding control concretes without the addition of ash (Figure 3).

B1 series concretes with a sand–gravel mixture of aggregates containing up to 30% ash additive achieved a higher average compressive strength than the control concrete. The highest compressive strength was achieved by B1-3 concretes containing 30% fly ash and it was approximately 24% higher than the strength obtained with the sample from the control series (B1-0). On the other hand, the addition of ash in the amount of 40% caused a decrease in the compressive strength of the tested concretes by approximately 3% compared to the control concrete. The compressive strength results for B2 series concretes with a basalt–sand mixture of aggregates and the addition of fly ash obtained after 7 days confirmed the tendency observed for the B1 series concretes. The increase in strength for the B2-3 series was 45% compared to the B2-0 control concrete. For concrete samples of the B3 series, in which fly ash was used as an additive to concrete made on the basis of CEM II/A-V 42.5 R cement, the highest compressive strength was also obtained by samples containing 30% fly ash, achieving an average compressive strength 28% higher than the B3-0 control series. The addition of fly ash from biomass increased the compressive strength of concrete from 12% to over 45%. The smallest standard deviation for samples tested after 7 days was 0.55 for samples from series B2-0 and the highest was 1.79 for samples from series B3-3.

**Figure 4:**
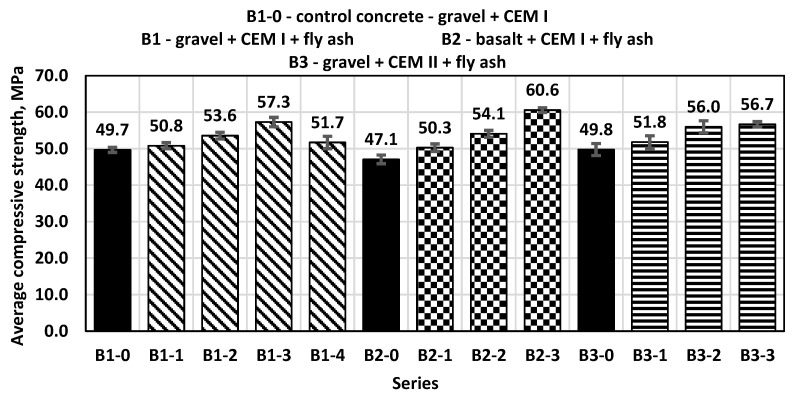
Average compressive strength after 28 days.

Compressive strength tests were also conducted for concrete samples after the standard time of 28 days of maturation. The control concrete (B1-0) with a sand–gravel mixture of aggregates obtained an average compressive strength of 49.7 MPa, while all concretes with the addition of fly ash in the amounts of 10%, 20%, 30% and 40% obtained higher compressive strength results than the control concrete (Figure 4).

The highest value of compressive strength was achieved by B1-3 concrete series with the addition of 30% fly ash as a substitute for part of the sand. As shown in the figure, the ash content at the level of 30% of the cement mass is the limit, leading to an increase in the compressive strength of concrete; higher amounts of added ash caused a decrease in the value of this parameter. The obtained test results for the B2 and B3 concrete series showed a similar tendency to concrete samples from the B1 series and increased this parameter by approximately 28%. The smallest standard deviation for samples tested after 28 days was 0.65 for samples from series B2-3 and the highest was 1.72 for samples from series B3-1.

**Figure 5:**
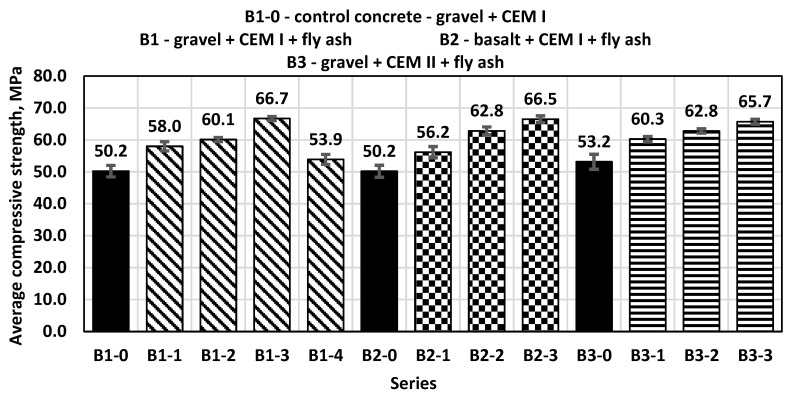
Average compressive strength after 56 days.

Compressive strength tests of concretes after 56 days of maturation were also conducted (Figure 5). At this stage, all concretes containing waste ash were marked by a higher compressive strength than the control concrete of the B1-0 series, and in the case of the B1 series of concrete, the increase in compressive strength was higher than in the case of the control concrete by 4–16% compared to the control concrete. The results obtained for concretes containing ash showed a similar tendency to concretes in the B2 and B3 series. Concretes with the addition of fly ash achieved higher compressive strengths than the control concrete, from 13 to 23%. Such a relationship may confirm that the ashes from the combustion of wood–sunflower biomass have a higher reactivity. The smallest standard deviation for samples tested after 56 days was 0.65 for samples from series B1-3 and the highest was 2.34 for samples from series B3-0.

### 3.3. The Impact of Ash Addition on the Water Absorption of Concrete

**Figure 6:**
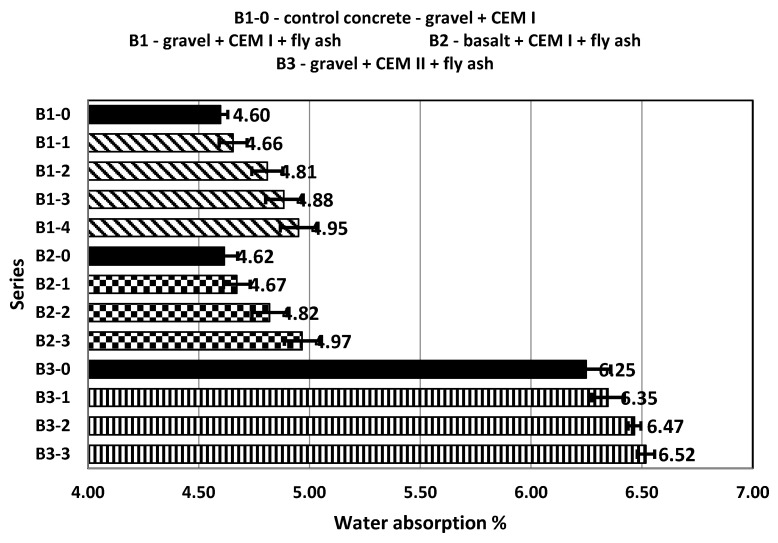
Water absorption of control concretes and concretes with the addition of fly ash.

Another property of the concretes tested was their water absorption, which was determined in accordance with the PN-B-06250 standard. Cubic samples with a side of 150 mm were prepared for the test. In the case of the B1-0 control concrete, the water absorption of the samples was 4.6%. The B1 series concretes, in which the fly ash from biomass combustion was added, showed a slightly higher water absorption, which increased along with the increase in the amount of added ash (Figure 6).

The B2 series concretes also showed an increase in water absorption along with the increase of the added ash, however, the difference between the control concrete and the concrete containing 30% ash added was only 0.35%. The water absorption of B3 series concretes, in which CEM II ash cement was used, was higher than in the case of concretes made with CEM I cement. Additionally, in these series of concretes, an increase in the water absorption of the tested concretes was observed along with an increase in the amount of added ash. The water absorption of concrete should not exceed 5% in the case of concretes directly exposed to atmospheric conditions and 9% in the case of concretes sheltered from direct atmospheric conditions. According to these guidelines, the B1-0 control concrete and all B1 and B2 series concretes could be used outside, while the B3 series concretes could be used inside. The smallest standard deviation for samples was 0.03 for samples from series B1-0 and the highest was 0.11 for samples from series B3-0.

### 3.4. The Impact of Ash Addition on Concrete Low-Temperature Resistance

**Figure 7:**
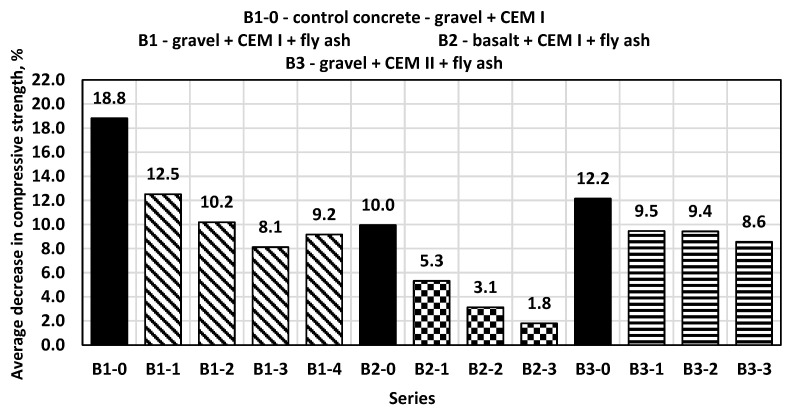
Decrease in the average compressive strength after frost resistance tests.

Low-temperature resistance tests of synthesized concretes were also conducted in accordance with the PN-B-06250:1988 standard. Closed pores play a key role in this, as they increase the resistance of concrete to freeze–thaw cycles. During freezing, ice is formed in open capillaries and the volume of the ice increases, the frozen water creating high hydrodynamic pressure and internal stresses. The decrease in compression strength in the control concretes after the freeze–thaw cycles was less than 19% (Figure 7).

In the case of the B1 series of concretes containing the addition of 10%, 20%, 30% and 40% of fly ash, a decrease in compressive strength was obtained by 12.5%, 10%, 8% and 9%, respectively. An addition of 30% fly ash can be considered optimal, due to this proportion being associated the lowest strength drop. All B1 series concrete samples had no visible damage or cracks after visual inspection. The low-temperature resistance test did not cause a large loss of mass, which was in the range of 0.01–0.03%. The B2 series concretes, in which crushed basalt aggregate was used, showed even smaller drops in compression strength after low-temperature resistance tests than the B1 series concretes. The addition of 30% of ash reduced the decrease in compression strength to a value of only 1.8%. The B3 series concretes also had no visible damage or cracks, and in all samples the use of fly ash was associated with a lower decrease in compression strength after low-temperature resistance tests. The best result was obtained for the concrete containing 30% of fly ash (strength decrease approximately 8.5%). To sum up, on the basis of the obtained test results, it can be concluded that the use of ashes from the combustion of wood–sunflower biomass in a fluidized bed boiler as substitutes for a part of the sand content in concrete reduces the decrease in the compression strength of concrete after low-temperature resistance tests. In each series of concrete containing ash, the decrease in compression strength was lower than for the control concrete. This addition reduced the decrease in compression strength by up to 80% compared to the control samples.

### 3.5. Water Penetration of Concretes Containing Ash from Biomass Combustion

**Figure 8:**
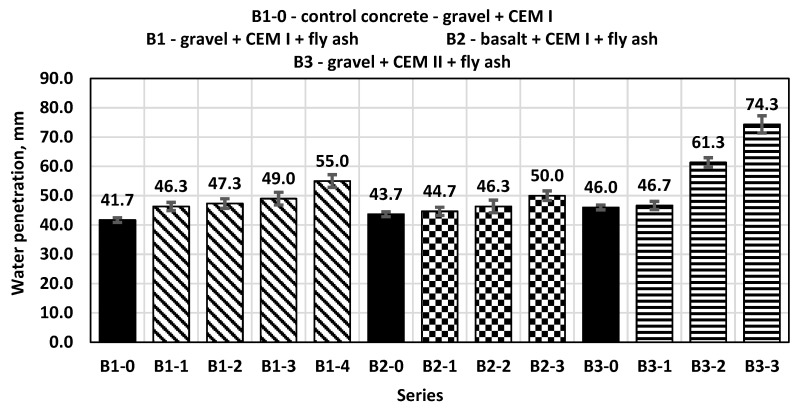
Water penetration of control concretes and concretes with the addition of fly ash.

The B1-0 control concrete, influenced by water pressure, obtained an average water penetration depth of 42 mm (Figure 8). The B1 series concretes were marked by a greater depth of water penetration, which increased with the increase in the amount of added ash. In the case of B2 and B3 series concretes, the trend observed for the B1 series concretes is visible. The lowest water penetration depth was achieved by the B2-0 and B3-0 control concretes, and the highest by those containing 30% fly ash. It can be noticed that in the B3 series concretes with CEM II ash cement, the increase in water penetration depth with increasing ash content increased to a greater extent and reached higher values than in the case of concretes made on the basis of CEM I cement.

### 3.6. Leaching Metal Ions from Concretes Containing Ash from Biomass Combustion

The test for the leaching of metal ions from concrete was conducted in accordance with the PN EN 12457 2:2006 standard. The leaching test was performed only for the control concrete (B1-0) and for concretes produced with the addition of fly ash in the amount of 30% of the cement mass (samples from the B1-3 series), assuming that the highest content in the eluate will occur for them. Two determinations were made for the tested concrete, maintaining the ratio of the volume of the liquid phase (L) to the mass of the solid phase (S) equal to 10:1. Calculations were made according to the standard assuming no moisture in the samples. After the process of leaching ions from the B1-0 control concrete, the pH of the solution was 10.10, while in the case of B1-3 concretes it was 9.67. The data presented in Table 5 show that the amount of leached toxic metal ions, such as Zn, Pb, Cu, Cr and Ba, from concretes containing fly ash (B1-3) is comparable to the amount of metal ions leached from the control concrete (B1-0). On the other hand, the content of toxic metal ions, such as Ni and Fe, from the concrete samples containing ash is slightly higher than from the control concrete samples. The obtained concentrations of Ni and Fe ions, as well as Zn, Pb, Cu, Cr and Ba metals, do not exceed the permissible values that must be met when discharging sewage into water or soil with substances particularly harmful to the environment, in accordance with the Regulation of the Minister of the Environment (Journal of Laws of 2014, item 1800). Thus, considering composite cracks and water penetration, concrete composites containing up to 30% fly ash do not pose a threat to the environment.

### 3.7. Microstructure of Composites with a Cement Matrix Containing Ashes from Biomass Combustion

**Figure 9:**
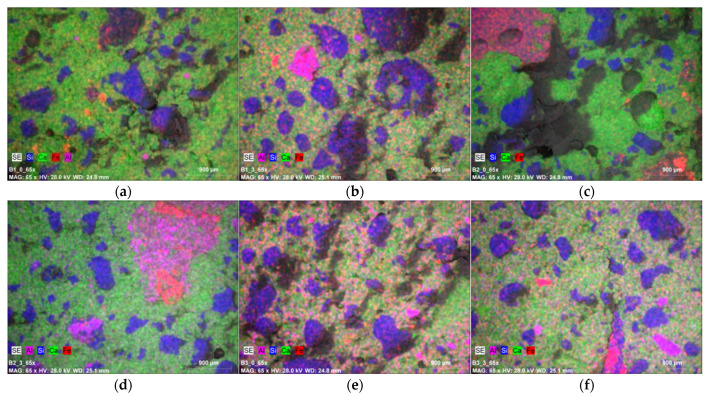
Maps of distribution of dominant elements in the studied area: (**a**) B1-0; (**b**) B1-3; (**c**) B2-0; (**d**) B2-3; (**e**) B3-0; (**f**) B3-3.

The tests were performed on broken surfaces of concrete samples. Figure 9 shows microscopic photos for the B1-0 concrete at 65× magnification and the X-ray energy dispersion analysis.

EDS analysis of the concrete surface performed in the area visible in the photo showed, apart from the presence of calcium (24.65%, green), a significant content of silicon (13.45%, blue), aluminium (2.31%; pink) and iron (2.72%, red). Sodium (1.56%) and potassium (0.39%) are present in lesser amounts. The remaining components (Mg, C, S, P) are present in a small amount (less than 0.7%). In the case of the B1-3 concrete samples containing 30% fly ash from biomass combustion, microscopic photos show a very similar structure to concrete without the addition of ash, which results from very fine granulation of the ash. The aggregate grains distributed evenly in the concrete are also clearly visible in these concretes. EDS analysis of the B1-3 concrete surface confirmed similar amounts of elements compared to the control concrete. In the B2-0 control concrete, in which sand was used as fine aggregate and a basalt mixture as coarse aggregate, microscopic photos showed basalt grains with irregular and sharp edges and the present fine aggregate with oval shapes; the largest share in the chemical composition is calcium (18.91%) and silicon (18.36%). In the case of the B2-3 concrete with 30% ash added, coarse basalt aggregate and quartz sand are also visible, and in terms of chemical composition, ash-free concrete (B2-0 series) and concrete with 30% fly ash added (B2-3 series) do not differ significantly from each other. The analysis of the B3 series surfaces showed that the microstructure of the B3-0 concrete, made on the basis of CEM II cement, has a very similar microstructure and appearance to the B1-0 concrete made on the basis of CEM I cement. The B3-3 concrete containing 30% fly ash under the microscope did not differ noticeably in structure from the B3-0 concrete. The EDS analysis of the B3-0 series concrete surfaces made on the basis of CEM II ash cement is also marked by a similar chemical composition. This concrete consists of approximately 23% calcium and 17% silicon, and similar results were obtained for samples with the addition of the B3-3 ash. The obtained results are not very precise guides, as only a few measuring points on the surface were anyalysed. In addition, the concrete structure is not homogenous throughout the sample volume. However, it can be concluded that non-destructive research methods using modern techniques for testing material structures are a way to effectively assess the impact of structure on the strength of concrete.

## 4. Conclusions

The use of fly ash from the wood–sunflower biomass combustion process in a circulating fluidized bed boiler for the production of building materials is a desirable solution in terms of sustainable construction.

1. The conducted tests of the properties and structure of composite materials with a cement matrix containing waste fluidized ashes from the combustion of wood–sunflower biomass (in the proportion of 80% wood waste and 20% sunflower waste) and the analysis of the obtained results enabled the conclusion that this ash, in the amount of 10% and up to 30%, can be added as a sand replacement for the production of composite materials with a cement matrix, such as concretes (made on the basis of CEM I or CEM II/A-V 42.5R cement), without reducing their quality (compression strength and low-temperature resistance) in relation to materials produced without the addition of waste ash.
2. The microstructure of concretes made on the basis of CEM I 42.5R cement containing fly ash from biomass combustion in the amount of 10–30% is marked by a microstructure very similar to the control concrete made without the addition of waste ash.
3. The addition of fly ash in the amount of 10–30% causes the concrete mix to condense and reduce its air content. The compression strength of concrete samples with sand–gravel and sand–basalt mixtures of aggregates made with the tested additive is higher than that obtained for the control samples.
4. The use of fly ash in the range of 10–30% causes a slight increase in the water absorption of concrete samples made with both sand–gravel and sand–basalt mixes of aggregates. The addition of waste ashes increases the depth of water penetration of the samples of all tested concrete series.
5. The addition of fly ash from biomass to concretes in amounts up to 30% reduces the decrease in compression strength of these concretes after low-temperature resistance tests and does not cause the formation of fragments, cracks and defects in the sample. In addition, concrete composites containing the addition of ash from biomass combustion, taking into account the crack of the composite and the penetration of water, do not show a negative impact on the natural environment related to the leaching (release) of heavy metals into the environment.
6. The use of waste ash has a positive effect on the natural environment by reducing the demand for natural resources. The use of fly ash in the amount of 30% of the cement mass as a sand substitute, depending on the type of aggregate mixture, reduces sand usage by 115–120 kg/m^3^, i.e., by about 15–20%.

## Figures and Tables

**Table 1:** Percentage of oxides and elements in fly ash.

Oxide/Element	Content %	Oxide/Element	Content %
SiO_2_	50.20	Na_2_O	0.44
CaO	11.82	MnO	0.28
K_2_O	7.99	TiO_2_	0.30
Al_2_O_3_	12.29	SO_3_	4.91
MgO	3.34	Cl	1.63
Fe_x_O_y_	3.50	Other	3.30

**Table 2:** Composition of B1-0, B2-0 and B3-0 control concretes.

Concrete	B1-0	B2-0	B3-0
Ingredient	Quantity		
Cement CEM I, kg	364.20	339.50	-
Cement CEM II, kg	-	-	364.20
Water, dm^3^	191.40	200.00	191.40
Aggregate—sand, kg	648.20	757.50	648.20
Aggregate G1; 2–8 mm, kg	662.00	-	662.00
Aggregate G2 8–16 mm, kg	541.70	-	541.70
Aggregate B1 2–8 mm, kg	-	668.90	-
Aggregate B2 8–16 mm, kg	-	547.30	-
Aggregate ∑, kg	1851.90	1973.70	1851.90
Plasticizer, dm^3^	1.82	2.72	1.82

**Table 3:** The composition of concretes with the addition of ash B1, B2 and B3 series.

Concrete	B1-1	B1-2	B1-3	B1-4	B2-1	B2-2	B2-3	B3-1	B3-2	B3-3
Ingredient	Quantity									
Cement CEM I, kg	364.20	364.20	364.20	364.20	339.47	339.47	339.47	-	-	-
Cement CEM II, kg	-	-	-	-	-	-	-	364.20	364.20	364.20
Water, dm^3^	191.40	191.40	191.40	191.40	200.00	200.00	200.00	191.40	191.40	191.40
Aggregate—sand, kg	607.10	566.00	525.00	484.00	719.20	680.90	642.70	607.10	566.00	525.00
Aggregate G1 2–8 mm, kg	662.10	662.10	662.10	662.10	-	-	-	662.10	662.10	662.10
Aggregate G2 8–16 mm, kg	541.70	541.70	541.70	541.70	-	-	-	541.70	541.70	541.70
Aggregate B1 2–8 mm, kg	-	-	-	-	668.90	668.90	668.90	-	-	-
Aggregate B2 8–16 mm, kg	-	-	-	-	547.30	547.30	547.30	-	-	-
Aggregate ∑, kg	1810.9	1769.8	1728.8	1687.8	1935.4	1897.1	1858.8	1810.9	1769.8	1728.8
Plasticizer, dm^3^	1.82	1.82	1.82	1.82	2.72	2.72	2.72	1.82	1.82	1.82
Fly ash, kg	36.40	72.80	109.20	145.60	33.95	67.89	101.84	36.40	72.80	109.20

**Table 4:** Consistency class and air content of B1, B2 and B3 concrete mixtures.

Series	Consistency Class B2-0		Air Content, %
Ingredient	mm	Class	
B1-0	145	S3	3.4
B1-1	95	S2/S3	3.4
B1-2	60	S2	2.9
B1-3	25	S1	2.7
B1-4	10	S1	2.5
B2-0	150	S3	4.0
B2-1	100	S3	3.7
B2-2	60	S2	2.9
B2-3	30	S1	2.4
B3-0	140	S3	3.7
B3-1	90	S2	3.2
B3-2	50	S2	2.6
B3-3	20	S1	2.4

**Table 5:** Results of the analysis of ion leaching from concrete samples.

Element	Sample				Limit Values
	B1-0		B1-3		
	A	s	A	s	
	mg/kg		mg/kg		mg/dm^3^
Zn	<0.005		<0.005		2
Cu	0.220	0.014	0.190	0.000	0.5
Ni	0.075	0.050	0.090	0.085	0.5
Ba	0.340	0.056	0.275	0.050	2
Pb	0.260	0.014	0.260	0.014	0.5
Cr	0.480	0.028	0.390	0.000	0.5
Fe	0.245	0.092	0.285	0.050	10
K	417.200	0.848	621.750	45.891	80
Na	184.900	29.699	186.550	8.273	800

A—released amount of component with L/S = 10, average based on two determinations; s—standard deviation based on two determinations.

## Data Availability

The data presented in this study are available on request from the corresponding author.
